# Support vector machine with hypergraph-based pairwise constraints

**DOI:** 10.1186/s40064-016-3315-x

**Published:** 2016-09-23

**Authors:** Qiuling Hou, Meng Lv, Ling Zhen, Ling Jing

**Affiliations:** College of Science, China Agricultural University, Beijing, 100083 China

**Keywords:** Support vector machine, Modified pairwise constraints, Hypergraph learning, Discrimination metric

## Abstract

Although support vector machine (SVM) has become a powerful tool for pattern classification and regression, a major disadvantage is it fails to exploit the underlying correlation between any pair of data points as much as possible. Inspired by the modified pairwise constraints trick, in this paper, we propose a novel classifier termed as support vector machine with hypergraph-based pairwise constraints to improve the performance of the classical SVM by introducing a new regularization term with hypergraph-based pairwise constraints (HPC). The new classifier is expected to not only learn the structural information of each point itself, but also acquire the prior distribution knowledge about each constrained pair by combining the discrimination metric and hypergraph learning together. Three major contributions of this paper can be summarized as follows: (1) acquiring the high-order relationships between different samples by hypergraph learning; (2) presenting a more reasonable discriminative regularization term by combining the discrimination metric and hypergraph learning; (3) improving the performance of the existing SVM classifier by introducing HPC regularization term. And the comprehensive experimental results on twenty-five datasets demonstrate the validity and advantage of our approach.

## Introduction

Support vector machine (SVM) (Vapnik 1995; Cortes and Vapnik 1995), founded on Vapnik’s statistical learning theory, has already reached many achievements in practical problems. For binary classification problems, its target is to find a separating hyperlane being the middle one between two parallel hyperplanes, where the two hyperplanes are constructed following the maximum margin principle. As for its solution, obtained by solving a quadratic programming problem (QPP) in the dual space, is global optimal. Furthermore, the kernel function (Shawe-Taylor and Cristianini 2004) introduced into SVM not only maps training vectors into a high-dimensional space, but also successfully transforms the nonlinear case into linear case. Thus, the case of nonlinear kernels is handled along lines similar to that used for linear kernels. Although the classical SVM has many good properties, one of the main challenges for it is the high computational complexity of the QPP. In addition, the trained performance also depends on the optimal parameters, which are usually found by cross-validation method. These shortcomings not only cause SVM to take a long time to train on a large database, but also prevent it from locating the optimal parameters from a very fine grid over a large span. Recently, many efficient learning algorithms and models related to SVM have emerged, such as the chunking algorithm (Cortes and Vapnik 1995), the decomposition method (Osuna et al. 1997), sequential minimal optimization (SMO) (Keerthi et al. 2001), the least squares support vector machine (LS-SVM) (Suykens et al. 1999), $\nu -$ SVM (Schölkopf et al. 2000), the generalized eigenvalue proximal support vector machine (GEPSVM) (Mangasarian and Wild 2006), and geometric algorithms (Franc and Hlavác 2003; Mavroforakis and Theodoridis 2007; Tao et al. 2008).

A common disadvantage of the existing large margin classifiers, including SVM, is that they fail to exploit the prior structural information which may be very important for classification effectiveness. In fact, for different problems, different classes may have different underlying data structural information. Thus, it is desirable that a classifier should be adaptable to the discriminant boundaries to fit the geometric structures of the data, especially for improving the generalization performance of the classifier. Recently, some efficient algorithms related to SVM have been developed to give more weightage to the structural information, which provide a novel view to design a classifier, i.e., a classifier should be sensitive to the prior structural distribution of the data (Yeung et al. 2007). Currently, there are mainly two strategies to design various algorithms based on the structural distribution of the training data. The first one is cluster assumption-based (Rigollet 2007), which assumes that the training data contains several clusters, and then deduces several popular large margin classifiers, such as ellipsoidal kernel machine (EKM) (Shivaswamy and Jebara 2007), minimax probability machine (MPM) (Lanckriet et al. 2002), maxi-min margin machine (M4) (Huang et al. 2004), and structured large margin machine (SLMM) (Yeung et al. 2007), structural regularized support vector machine (SRSVM) (Xue et al. 2011). The second strategy is manifold assumption-based, which assumes that the training data actually lies on a low-dimensional submanifold in the input space. A typical paradigm in this strategy is Laplacian support vector machine (Lap-SVM) (Belkin et al. 2004), which constructs a Laplacian graph for each class by exploiting the local neighborhoods of each data to form the corresponding Laplacian matrix to reflect the geometric structure of each class data. And then they are embedded into the traditional SVM framework as additional manifold regularization terms.

Even though the above modified SVM methods utilize the prior structural information of the training data to adjust the discriminant boundaries, there still might be some useful knowledge neglected, for example, the additional regularization term only indicates the relationship between two corresponding samples, without considering the high-order relationship between several samples.

The traditional pairwise constraints (PC) method (Hu et al. 2008; Yu et al. 2012a, b; Qian et al. 2013), which is powerful in semi-supervised or unsupervised learning tasks, mainly pays attention to the discrimination distance between two patterns while neglecting the spatial distance that might be also important. To overcome this drawback (Zhu et al. 2015), designed a new strategy to combine the discrimination metric based on the traditional PC and the Euclidean distance measure together, i.e., the modified pairwise constraints (MPC) method. In MPC, the spatial measure strategy is based on the simple-graph-constructing. However, the simple graph learning methods only consider the pairwise relationship between two samples and ignore the high-order relationship between several samples. Hypergraph learning (Zhou et al. 2006; Yu et al. 2012a, b; Wei et al. 2015) aims to get the relationship between several samples in a higher order, and thus achieves a promising performance in many applications. Inspired by the above studies, we design a novel hypergraph-based pairwise constraints (HPC) regularization term, which not only acquires the discriminative information about each constrained pair, but also considers the higher order relationship between different patterns. In this paper, we introduce the newly-designed HPC regularization term into SVM, and present a novel algorithm, i.e., support vector machine with hypergraph-based pairwise constraints (HPCSVM). This HPCSVM not only retains the superior characteristics of SVM, but also has its additional advantages: (1) getting comparable or better classification accuracies compared to SVM and its variants; (2) acquiring high-order relationship between several samples by hypergraph learning; (3) presenting a more reasonable discriminative regularization term by combining the discrimination metric and the hypergraph learning.

The rest of this paper is organized as follows. In Background” section, brief overviews of SVM and hypergraph learning are given. In “Support vector machine with hypergraph-based pairwise constraints” section, we first introduce the newly-designed HPC regularization term, and then detail the proposed HPCSVM, both the linear and nonlinear cases are included. “Experiments” section discusses the comprehensive experimental results on the UCI benchmark datasets to investigate the feasibility and validity of our proposed algorithm, and “Conclusion” section concludes the paper.

## Background

### Support vector machine

SVM is a powerful and promising paradigm for pattern classification and regression. It emerges from research in statistical learning theory about how to regulate the trade-off between empirical risk and structural complexity. And its main attempt is to reduce the generalization error by maximizing the margin between two parallel supporting hyperplanes.

Given a training dataset $T = \{ (x_{1} ,y_{1} ), \ldots ,(x_{l} ,y_{l} )\}$, where $x_{i} \in R^{n}$, $y_{i} \in \{ + 1, - 1\}$, $i = 1,2, \ldots ,l$. SVM searches for an optimal separating hyperplane to correctly separate the positive points and the negative points defined as

1 $$(w \cdot x) + b = 0$$

where $$w \in R^{n}$$ and $$b \in R$$.

By introducing the regularization term $\frac{1}{2}\left\| w \right\|^{2}$ and the slack variable $\xi = (\xi_{1} ,\xi_{2} , \cdots ,\xi_{l} )^{T}$, the optimization problem corresponding to SVM can be expressed as

2 $$\begin{aligned} & \mathop {\hbox{min} }\limits_{w,b,\xi } {\kern 1pt} \,\frac{1}{2}||w||^{2} + C\sum\limits_{i = 1}^{l} {\xi_{i} } \\ & s.t.\quad y_{i} ((w \cdot x_{i} ) + b) \ge 1 - \xi_{i} \\ & \xi_{i} \ge 0,\quad i = 1, \ldots ,l. \\ \end{aligned}$$

where $C > 0$ is a penalty parameter. Note that the minimization of the regularization term $\frac{1}{2}\left\| w \right\|^{2}$ is equivalent to the maximization of the margin between two classes. Generally, rather than solving (2), we solve its dual problem to get the appropriate margin classifier.

Using the dual optimization technique, one can show that the dual problem of (2) can be expressed as

3 $$\begin{aligned} & \mathop {\hbox{min} }\limits_{\alpha } {\kern 1pt} \,\frac{1}{2}\sum\limits_{i = 1}^{l} {\sum\limits_{j = 1}^{l} {\alpha_{i} \alpha_{j} y_{i} y_{j} (x_{i} \cdot x_{j} )} } - \sum\limits_{j = 1}^{l} {\alpha_{j} } \\ & s.t.\quad \sum\limits_{i = 1}^{l} {y_{i} \alpha_{i} } = 0, \\ & 0 \le \alpha_{i} \le C, \quad i = 1, \cdots l. \\ \end{aligned}$$

where $\alpha_{i}$, $i = 1, \ldots ,l,$ are the Lagrangian multipliers.

Suppose the solution of (3) is $\alpha^{*} = (\alpha_{1}^{*} , \cdots ,\alpha_{l}^{*} )^{T}$, then

4 $$w^{*} = \sum\limits_{i = 1}^{l} {\alpha_{i}^{*} y_{i} x_{i} }$$

5 $$b^{*} = y_{j} - \sum\limits_{i = 1}^{l} {\alpha_{i}^{*} y_{i} (x_{i} \cdot x_{j} )}$$

where $\alpha_{j}^{*}$ is a component of $\alpha_{{}}^{*}$, and $\alpha_{j}^{*} \in (0,C)$.

A new sample $x_{{}}^{{}}$ is classified as +1 or −1 according to the final decision function as follows

6 $$f(x) = \text{sgn} \left( {\sum\limits_{i = 1}^{l} {\alpha_{i}^{*} y_{i} (x_{i} \cdot x)} + b^{*} } \right)$$

### Hypergraph learning

**Fig. 1:**
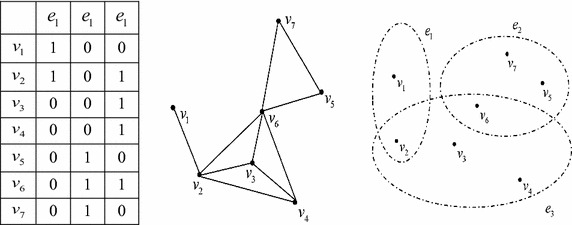
Simple graph versus hypergraph. *Left*: an article set $V = \{ v_{1} ,v_{2} ,v_{3} ,v_{4} ,v_{5} ,v_{6} ,v_{7} \}$ and an author set $E = \{ e_{1} ,e_{2} ,e_{3} \}$. The element $(v_{i} ,e_{j} )$ is set to 1 if $e_{j}$ is an author of article $v_{i}$, and 0 otherwise. Middle: an undirected simple graph in which two articles are joined together by an edge if there is at least one author in common. While, this graph cannot tell us whether the same person is the author of three or more articles or not. *Right*: a hypergraph which could completely illustrate the high-order relationships among authors and articles

Hypergraph learning is derived from the theory of simple graph learning. In a simple graph, an edge is connected with two samples and the weight of the edge only indicates the relationship between two corresponding samples. While in reality, the high-order relationship between several samples is critical. Thus we can completely represent the complex relationships among samples by using hypergraph, in which each hyperedge could connect more than two samples. Below we give out a concrete example (Fig. 1) to show the difference between simple graph and hypergraph.

Given a dataset $V = \{ x_{1} , \ldots ,x_{l} \}$, where $x_{i} \in R^{n}$, $i = 1,2, \ldots ,l$. Hypergraph $G = (V,E,W)$ is composed of the vertex set $V_{{}}$ and the hyperedge set $E$. $W_{{}}$ is a diagonal matrix with its diagonal elements indicating the weights of the hyperedges. Each hyperedge $e$ is a subset of $V$, and the weight of hyperedge $e$ is denoted as $w(e)$. The incidence matrix $\varphi_{{|V| \times |E{\kern 1pt} |}}$ of $G_{{}}$ is defined as follows

7 $$\varphi (v,e) = \left\{ {\begin{array}{ll} 1 & \quad {if \quad v \in e} \\ 0 & \quad {if \quad v \notin e} \\ \end{array} } \right.$$

The degree of a vertice $v$ is defined as

8 $$d(v) = \sum\limits_{{\{ e \in E|v{\kern 1pt} \in e\} }} {w(e)} = \sum\limits_{e \in E} {w(e)\varphi (v,e)}$$

The degree of a hyperedge $e$ is defined as

9 $$\delta (e) = |e| = \sum\limits_{v \in V} {\varphi (v,e)}$$

The weight of a hyperedge $e$ is defined as

10 $$w(e) = {\kern 1pt} \sum\limits_{{x_{j} \in e}} {dist(x_{e} ,x_{j} )}$$

where $x_{e}$ is the centroid vertex of the hyperedge $e$.

And the distance between two samples is

11 $$dist(x_{i} ,x_{j} ) = \exp \left( { - \frac{{||x_{i} - x_{j} ||^{2} }}{{\sigma^{2} }}} \right)$$

where

12 $$\sigma = \sqrt {\frac{1}{l - 1}\sum\limits_{i = 1}^{l} {||x_{i} - \bar{x}||^{2} } } , \quad \bar{x} = \frac{1}{l}\sum\limits_{i = 1}^{l} {x_{i} } .$$

Then, the adjacency matrix $P_{{}}$ of the hypergraph $G_{{}}$ is defined as $P = \varphi WD_{e}^{ - 1} \varphi^{T}$, where $D_{e}$ is a diagonal matrix with its diagonal elements indicating the degrees of hyperedges, $\varphi^{T}$ is the transpose of $\varphi$.

## Support vector machine with hypergraph-based pairwise constraints

In theory, the connection between the intra-class patterns should be as strong as possible, while the connection between inter-class patterns should be as weak as possible. In this section, we present an HPC strategy to strengthen the connection of intra-class patterns and weaken the connection of inter-class patterns, and then introduce this novel trick into SVM. More specifically, on the one hand, the relationships between several samples in a higher order are acquired by hypergraph learning, on the other hand, a more reasonable discriminative regularization term is presented by combining the discrimination metric and hypergraph learning, at last, this novel regularization term is introduced into SVM to get a better classifier.

### Hypergraph-based pairwise constraints

This subsection first introduces the previous work of PC, and then presents an MPC strategy to overcome the drawback of PC without acquiring the spatial distribution of samples, finally, an HPC strategy combining hypergraph learning is proposed.

#### The traditional pairwise constraints method

PC, i.e., the must-link and cannot-link constraints, were first introduced in Lange et al. (2005). In detail, the must-link means a pair of samples should be allotted to the same cluster, while the cannot-link performs the opposite operation. Since then, efforts have been made to apply PC to both clustering (Hu et al. 2008; Zeng et al. 2012; Qian et al. 2013) and supervised classification (Ko et al. 2007; Li et al. 2012) tasks. Besides, it is also utilized for semi-supervised classification (Goldberg et al. 2007; Zhang et al. 2010), the feature extraction (Sun and Zhang 2010; Yang and Song 2010), the dimension reduction (Wang et al. 2010), and the neural network (Maggini et al. 2012).

The form of the traditional PC discussed in this paper goes along the same lines as that was defined in (Qian et al. 2013; Ko et al. 2007; Li et al. 2012)

13 $$|f(x_{i} ) - z_{ij} f(x_{j} )|$$

where $x_{i}$ and $x_{j}$ are two samples, whose labels are $y_{i}$ and $y_{j}$, respectively, and $z_{ij} = y_{i} y_{j}$. This form gives out a discrimination metric to strengthen the connection of intra-class samples and weaken the connection of inter-class samples, which plays a similar role as the Laplacian loss in (Hu et al. 2008).

#### Modified pairwise constraints method

The traditional PC method might lose its efficacy when dealing with classification problem because it learns the relationship between each pair of samples from the prediction function without acquiring the spatial distribution of samples. To overcome this drawback (Zhu et al. 2015), combined the spatial information and the traditional PC and presented a modified PC as

14 $$m_{pc} = w_{ij} ||f(x_{i} ) - z_{ij} f(x_{j} )||^{2}$$

where $x_{i}$ and $x_{j}$ are two samples, whose labels are $y_{i}$ and $y_{j}$ respectively, $z_{ij} = y_{i} y_{j}$, and the spatial measure strategy is adopted as

15 $$w_{ij} = \left\{ {\begin{array}{*{20}l} {\exp \left( { - \frac{{||x_{i} - x_{j} ||^{2} }}{\gamma t}} \right)} \hfill & \quad {if\quad x_{j} \in N_{k} (x_{i} )\,or\,x_{i} \in N_{k} (x_{j} )} \hfill \\ 0 \hfill & \quad {otherwise} \hfill \\ \end{array} } \right.$$

where, $N_{k} (x_{i} )$ contains the k-nearest neighbors of the sample $x_{i}$, $\gamma_{{}}$ is the coefficient to control the influence of $t_{{}}$, and $t_{{}}$ is the mean squared mutual distance between each pair samples.

In MPC, the weight $w_{ij}$ is relatively large when the two samples are spatially close to each other, while $w_{ij}$ becomes relatively small when the two samples are spatially away from each other, which is the most commonly used spatial distribution learning strategy.

#### Hypergraph-based pairwise constraints method

In MPC, the spatial measure strategy is essentially based on simple graph. So, the weight only indicates the relationship between the two corresponding samples. While in reality, relationships among the samples of our interest are more complex than pairwise. Therefore, we consider using hypergraph instead of the simple graph to completely represent the complex relationships among the samples.

Extract the vertex set $V = \{ x_{1} , \ldots ,x_{l} \}$ from the given training set $T = \{ (x_{1} ,y_{1} ), \ldots ,(x_{l} ,y_{l} )\}$, then establish a hypergraph $G = (V,E,W)$ as in “Hypergraph learning” section. The adjacency matrix $P$ of hypergraph $G_{{}}$ is defined as $P = \varphi WD_{e}^{ - 1} \varphi^{T}$, where $\varphi ,W,D_{e}$ represent the same meaning as in “Hypergraph learning” section. We present the hypergraph-based PC as follows

16 $$h_{pc} = p_{ij} ||f(x_{i} ) - z_{ij} f(x_{j} )||^{2}$$

where $x_{i}$ and $x_{j}$ are two samples, whose labels are $y_{i}$ and $y_{j}$, respectively, and $z_{ij} = y_{i} y_{j}$.

To be more feasible and effective, here we adopt the distance measure strategy as follows

17 $$dist(x_{i} ,x_{j} ) = \exp \left( { - \frac{{||x_{i} - x_{j} ||^{2} }}{{\gamma \sigma^{2} }}} \right)$$

where $\gamma_{{}}$ is the coefficient to control the influence of $\sigma_{{}}^{2}$, and

18 $$\sigma^{2} = \frac{1}{l - 1}\sum\limits_{i = 1}^{l} {||x_{i} - \bar{x}||^{2} } {\kern 1pt} , \quad \bar{x} = \frac{1}{l}\sum\limits_{i = 1}^{l} {x_{i} .}$$

### Linear case

SVM aims to find an optimal separating hyperplane, which is defined as $f(x) = (w \cdot x) + b = 0$, to correctly separate the two class points. According to Formula (16), the hypergraph-based regularization term $R_{pc}$ is formulated as

19 $$R_{pc} = \frac{1}{2}\sum\limits_{i = 1}^{l} {\sum\limits_{j = 1}^{l} {p_{ij} ||f(x_{i} ) - z_{ij} f(x_{j} )||^{2} } } = f^{T} L^{{\prime }} f$$

where $f = [f(x_{1} ),f(x_{2} ), \ldots ,f(x_{l} )]^{T} = Mw + sb$, $M \in R_{{}}^{l \times n}$ includes all of the samples, $s$ is a vector of ones of appropriate dimensions, $L^{\prime} = L + (U - Z) \cdot P$, $L_{{}} = D - P$, $D$ is a diagonal matrix with $d_{ii} = \sum\nolimits_{j = 1}^{l} {p_{ij} }$, $U$ is a all-one matrix and $\cdot$ is the Hadamard product. Similarly, we can also formulate the regularization term corresponding to the negative hyperplane.

By adding the proposed regularization term $R_{pc}$ into SVM, we construct the optimization problems as follows:

20 $$\begin{aligned} & \mathop {\hbox{min} }\limits_{w,b,\xi } \frac{1}{2}||w||^{2} + \frac{{c_{1} }}{2}\sum\limits_{i = 1}^{l} {\sum\limits_{j = 1}^{l} {p_{ij} ||((w \cdot x_{i} ) + b) - z_{ij} ((w \cdot x_{j} ) + b)||^{2} } } + c\sum\limits_{i = 1}^{l} {\xi_{i} } \\ & s.t.\quad y_{i} ((w \cdot x_{i} ) + b) \ge 1 - \xi_{i} , \\ & \xi_{i} \ge 0,\quad i = 1,2, \ldots ,l. \\ \end{aligned}$$

where $c,c_{1} > 0$ are the parameters used to denote the trade-off among each term in the objective function, $\xi_{i} ,i = 1, \ldots ,l,$ are the slack variables, and $s$ is a vector of ones of appropriate dimensions.

The first term of (20) has the same effect as in SVM, i.e., maximizing the margin between two disjoint half planes. The second term is the discriminative information regularization term, which exploits the similarity and the dissimilarity of the labels through utilizing pairwise constraint information. If the two items belong to the same class, we encode the similarity between $x_{i}$ and $x_{j}$ as $p_{ij} (f(x_{i} ) - f(x_{j} ))^{2}$, and minimization of this penalty term tends to compel $f(x_{i} ) \approx f(x_{j} )$, i.e., the examples which have the same label should have the approximate real values. If the two items belong to different classes, we encode the dissimilarity between $x_{i}$ and $x_{j}$ as $p_{ij} (f(x_{i} ) + f(x_{j} ))^{2}$, and minimization of this penalty term tends to compel $f(x_{i} ) \approx - f(x_{j} )$, i.e., the real values of the examples which have different labels should be close to a pair of opposite number. And $p_{ij}$ is the weight between two patterns, which implies the relationship between several samples in a higher order. The third term is the empirical risk, which restricts that negative samples should lie below the bounding plane $(w \cdot x) + b = - 1$, while positive samples should lie above the bounding plane $(w \cdot x) + b = 1$.

In order to solve the problem (20), we construct its matrix form

21 $$\begin{aligned} & \mathop {\hbox{min} }\limits_{w,b,\xi } \frac{1}{2}||w||^{2} + \frac{{c_{1} }}{2}(Mw + sb)^{T} L^{{\prime }} (Mw + sb) + cs^{T} \xi \\ & s.t.\quad y \cdot (Mw + sb) \ge s - \xi , \\ & \xi \ge 0. \\ \end{aligned}$$

where $y$ is a label vector of all the samples, and $\cdot$ is the Hadamard product.

By introducing the Lagrangian function of (21)

22 $$L = \frac{1}{2}||w||^{2} + \frac{{c_{1} }}{2}(Mw + sb)^{T} L^{\prime}(Mw + sb) + cs^{T} \xi - \alpha^{T} (y \bullet (Mw + sb) - s + \xi ) - \beta^{T} \xi {\kern 1pt}$$

where $\alpha = (\alpha_{1} ,\alpha_{2} , \ldots ,\alpha_{l} )^{T}$ and $\beta = (\beta_{1} ,\beta_{2} , \ldots ,\beta_{l} )^{T}$ are the Lagrange multiplier vectors. And the Karush–Kuhn–Tucker (K.K.T) conditions for (22) are given by

23 $$\frac{\partial L}{\partial w} = w + c_{1} M^{T} L^{{\prime }} (Mw + sb) - (y \circ M)^{T} \alpha = 0,$$

24 $$\frac{\partial L}{\partial b} = c_{1} s^{T} L^{{\prime }} (Mw + sb) - y^{T} \alpha = 0,$$

25 $$\frac{\partial L}{\partial \xi } = cs - \alpha - \beta = 0,$$

26 $${\kern 1pt} y \cdot (Mw + sb) \ge s - \xi ,\quad \xi \ge 0,$$

27 $$\alpha^{T} (y \cdot (Mw + sb) - s + \xi ) = 0 \quad \beta^{T} \xi = 0,$$

28 $$\alpha \ge 0,\beta \ge 0.$$

where $y \circ M$ defines a matrix of the same size as $M$, of which the $ith$ row is $y_{i} \cdot M_{i}$.

Since $\beta \ge 0$, from (25) we have

29 $$0 \le \alpha \le cs.$$

Next, combining (23) and (24) leads to

30 $$\left( {\begin{array}{*{20}c} I & \quad \\ & \quad 0 \\ \end{array} } \right)\left[ {\begin{array}{*{20}c} w \\ b \\ \end{array} } \right] + c_{1} \left[ {\begin{array}{*{20}c} {M^{T} } \\ {s^{T} } \\ \end{array} } \right]L^{\prime}[M{\kern 1pt} {\kern 1pt} {\kern 1pt} {\kern 1pt} {\kern 1pt} s]\left[ {\begin{array}{*{20}c} w \\ b \\ \end{array} } \right] - (y \circ J)^{T} \alpha = 0.$$

Define $$H = \left( {\begin{array}{*{20}l} I \hfill & \quad \hfill \\ \hfill & \quad 0 \hfill \\ \end{array} } \right)$$, $J = [M{\kern 1pt} {\kern 1pt} {\kern 1pt} \;s]$. The Eq. (30) can be rewritten as

31 $$(H + c_{1} J^{T} L^{{\prime }} J)\left[ {\begin{array}{*{20}c} w \\ b \\ \end{array} } \right] - (y \circ J)^{T} \alpha = 0,$$

Then

32 $$\left[ {\begin{array}{*{20}c} w \\ b \\ \end{array} } \right] = (H + c_{1} J^{T} L^{{\prime }} J)^{ - 1} (y \circ J)^{T} \alpha .$$

To avoid the positive semi-definite matrix $H + c_{1} J^{T} L^{{\prime }} J$ being irreversible, a regularization term $\varepsilon I(\varepsilon > 0)$ is introduced. Then, (32) gets modified to the following formulation

33 $$\left[ {\begin{array}{*{20}c} w \\ b \\ \end{array} } \right] = (H + c_{1} J^{T} L^{{\prime }} J + \varepsilon I)^{ - 1} (y \circ J)^{T} \alpha .$$

Finally, the Wolfe’s dual of (21) is derived as follows

34 $$\begin{aligned} & \mathop {\hbox{max} }\limits_{\alpha } \quad s^{T} \alpha - \frac{1}{2}\alpha^{T} (y \circ J)(H + c_{1} J^{T} L^{{\prime }} J)^{ - 1} (y \circ J)^{T} \alpha \\ & s.t.\quad 0 \le \alpha_{i} \le c, \quad i = 1,2, \ldots ,l.{\kern 1pt} {\kern 1pt} \\ \end{aligned}$$

Suppose the solution of (34) is $\alpha^{*} = (\alpha_{1}^{*} , \ldots ,\alpha_{l}^{*} )^{T}$, then the augmented vector of (32) can be obtained. And a new testing sample $x_{{}}^{{}}$ is classified as +1 or −1 according to the final decision function as follows

35 $$f(x) = \text{sgn} ((w^{*} \cdot x) + b^{*} )$$

### Nonlinear case

In order to extend our HPCSVM to the nonlinear case, we rewrite the decision function as:

36 $$f(x) = (w \cdot \Phi (x)) + b = 0$$

where $\Phi ( \cdot )$ is a nonlinear mapping from a low dimensional space to a higher dimensional Hilbert space. According to Hilbert space theory (Schölkopf and Smola 2002), $w$ can be expressed as $w = \sum\nolimits_{i = 1}^{l} {u_{i} \Phi (x_{i} )}$. So the decision function can be expressed as:

37 $$f(x) = K(x^{T} ,M^{T} )u + b = 0$$

where $K( \cdot )$ stands for a kernel function: $K(x_{i} ,x_{j} ) = (\varPhi (x_{i} ) \cdot \varPhi (x_{j} ))$. So the nonlinear optimization problems can be expressed as

38 $$\begin{aligned} & \mathop {\hbox{min} }\limits_{u,b,\xi } \frac{1}{2}u^{T} Ku + \frac{{c_{1} }}{2}\sum\limits_{i = 1}^{l} {\sum\limits_{j = 1}^{l} {p_{ij} ||(K(x_{i}^{T} ,M^{T} )u + b) - z_{ij} (K(x_{j}^{T} ,M^{T} )u + b)||^{2} } } + c\sum\limits_{i = 1}^{l} {\xi_{i} } \\ & s.t.\quad y_{i} (K(x_{i}^{T} ,M^{T} )u + b) \ge 1 - \xi_{i} , \\ & \xi_{i} \ge 0, \quad i = 1,2, \ldots ,l. \\ \end{aligned}$$

where $c,c_{1} > 0$ are the predefined parameters, $\xi_{i} ,i = 1, \ldots ,l,$ are the slack variables, $s$ is a vector of ones of appropriate dimensions, and $M \in R_{{}}^{l \times n}$ includes all of the samples.

Similarly, we rewrite the matrix form of (38) as follows

39 $$\begin{aligned} & \mathop {\hbox{min} }\limits_{u,b,\xi } \frac{1}{2}u^{T} Ku + \frac{{c_{1} }}{2}(Ku + sb)^{T} L^{{\prime }} (Ku + sb) + cs^{T} \xi \\ & s.t.\quad y \cdot (Ku + sb) \ge s - \xi , \\ & \xi \ge 0. \\ \end{aligned}$$

where $$K = K(M,M^{T} )$$.

Adopting the similar process to the linear case, we can derive the dual formulation of (39) as follows

40 $$\begin{aligned} & \mathop {\hbox{max} }\limits_{\alpha } \quad s^{T} \alpha - \frac{1}{2}\alpha^{T} (y \circ J_{\varPhi } )(H_{\varPhi } + c_{1} J_{\varPhi }^{T} L^{{\prime }} J_{\varPhi } )^{ - 1} (y \circ J_{\varPhi } )^{T} \alpha \\ & s.t.\quad 0 \le \alpha_{i} \le c,\;i = 1,2, \ldots ,l.{\kern 1pt} {\kern 1pt} \\ \end{aligned}$$

where $y$ is a label vector of all the samples, $$H_{\varPhi } = \left( {\begin{array}{*{20}c} K & {} \\ {} & 0 \\ \end{array} } \right)$$, $J_{\varPhi } = [K{\kern 1pt} {\kern 1pt} {\kern 1pt} \;s]$.

Furthermore, we can get

41 $$\left[ {\begin{array}{*{20}c} u \\ b \\ \end{array} } \right] = (H_{\varPhi } + c_{1} J_{\varPhi }^{T} L^{{\prime }} J_{\varPhi } + \varepsilon I)^{ - 1} (y \circ J)^{T} \alpha .$$

Once the augmented vector of (41) is obtained, a new testing sample $x_{{}}^{{}}$ is classified as +1 or −1 according to the final decision function as follows

42 $$f(x) = \text{sgn} (K(x^{T} ,M^{T} )u^{*} + b^{*} )$$

### Analysis of algorithm

According to statistical theory, the training points are generated independently and identically according to an unkow but fixed probability distribution, i.e., all the training points should have some degree of underlying correlation. However, in SVM and its many variants, the potential structural information of the training data has not been taken into account when constructing optimization problems. In this paper, we present a novel discriminative regularization term named $R_{pc}$ with hypergraph-based PC method, which is expected to acquire the prior distribution knowl- edge about each constrained pair with both the discrimination metric from the traditional PC and the high-order relationship between different samples from hypergraph learning, and then introduce it into SVM.

Now we analyze our proposed HPCSVM concretely:

1. Inheriting the maximal-margin principle. In SVMs, the minimization of the regularization term $\frac{1}{2}\left\| w \right\|^{2}$ is equivalent to the maximization of the margin between the two parallel supporting hyperplanes. In our HPCSVM, we still choose the same regularization term $\left\| w \right\|^{2}$ to reflect the capacity of the decision function and the size of margin.
2. Extracting the potential structural information of the data. In SVM and its many variants, the optimal separating hyperplane is established with considering each sample independently, i.e., without considering the relationship between every pair of samples. In our algorithm, we design a novel HPC regularization term to extract the discriminative information about each constrained pair as the potential structural information, and then apply it to adjust the separating hyperplane.
3. Getting the relationship between several samples in a higher order. In MPC, the spatial measure strategy is essentially based on simple graph. While in reality, relationships among the samples are more complex than pairwise. Therefore, we use a hypergraph instead of the simple graph to completely represent the complex relationships among the samples and propose a novel discriminative information regularization term named HPC.
4. The limitation of our HPCSVM. A limitation of HPCSVM is that it cannot handle large-scale problems. There are two main reasons leading to such a limitation. On the one hand, our HPCSVM has to find the k-nearest neighbors for all the samples in the stage of the establishment of hypergraph. On the other hand, the computation and storage of kernel function are the bottlenecks of almost all SVMs, so does the HPCSVM.

## Experiments

In this section, we demonstrate the validity and efficiency of our proposed method HPCSVM on twenty-five benchmark datasets from UCI machine learning repository by comparing with SVM, LSSVM, structural regularized support vector machine (SRSVM) and support vector machine with modified pairwise constraints (MPCSVM). To make the results more convincing, we use five-fold cross-validation (Duda et al. 2001) to estimate the accuracy of each experiment. More specifically, the training set is randomly partitioned into five subsets which are roughly of equal size, and one of those subsets is reserved as the testing set whereas the remaining subsets serve as the training set. This process is repeated five times until all of the five subsets have been set to be a testing one once, and the average of the five accuracies is regarded as the classification accuracy of each experiment.

All the algorithms are written in MATLAB 2012a on Windows 7 running on a PC with system configuration Intel(R) Core(TM) 2 Duo CPU E7500 (2.93 GHz) with 2.00 GB of RAM. And the evaluation criterion of each algorithm is the classification accuracy of the testing examples, which is defined as follows:

43 $$Acc = \frac{TP + TN}{TP + FP + TN + FN}$$

where $TP$, $TN$, $FP$ and $FN$ are the numbers of true positive, true negative, false positive and false negative on the testing examples, respectively.

### Parameter selection

In our experiments, we adopt the grid search method to get the optimal parameters. In addition, for the nonlinear case, all the algorithms adopt Gaussian kernel $K(x,y) = \exp ( - \sigma ||x - y||^{2} )$ for the decision space. As for the tuning parameters, i.e., the Gaussian kernel parameter $\sigma$, the penalty parameter $c$, and the trade-off parameter $c_{1}$ are all selected from the set: $\{ 10^{ - 2} ,10^{ - 1} ,10^{0} ,10^{1} ,10^{2} \}$. And the optimal value $k_{{}}$ for k-nearest neighbors in MPCSVM and HPCSVM is searched from the set: $\{ 3,4,5,6,10,15\}$. For large-scale problems, the range of all the parameters will be narrowed uniformly due to the long training time.

### Experimental results and discussions

We experiment our HPCSVM on twenty-five real-world datasets from the UCI machine learning repository. These datasets represent a wide range of fields (include pathology, finance, agronomy and so on), sizes (from 100 to 1473) and features (from 3 to 60). All the datasets are normalized such that the feature’s scale is in [0, 1] before training.

#### Comparisons of different methods

In order to prove our proposed method be better, we compare the experimental results of various methods mentioned above. The main objects and motivations of the comparison are shown in Table 1.

**Table 1:** The objects and motivations of the comparison

Situation	Objects	Motivations
1	SRSVM versus SVM	To demonstrate the prior structural information within classes in the data is effective for classification
2	MPCSVM versus SVM	To demonstrate the discriminative information about each constrained pair in data is effective for classification
3	MPCSVM versus SRSVM	To display the discriminative information is more effective than the structures in data within classes for classification
4	HPCSVM versus MPCSVM	To demonstrate our newly-designed HPC regularization term is more reasonable than the MPC regularization term
5	HPCSVM versus LSSVM	To display our proposed HPCSVM is also better than SVM’s variant

#### Result comparisons and discussion

This subsection describes empirical comparisons of various models, such as SVM, LSSVM, SRSVM, MPCSVM and HPCSVM. The average classification accuracies and standard deviations are reported in Tables 2 and 3. And the average execution time of five-fold cross-validation for each experiment is demonstrated too. Complementally, for MPCSVM and HPCSVM, the execution time includes graph establishment. The comparison results in italic face are the best results. Table 2 shows the performance of linear HPCSVM with that of linear SVM, LSSVM, SRSVM and MPCSVM. Table 3 gives the performance of nonlinear HPCSVM with that of nonlinear SVM, LSSVM, SRSVM and MPCSVM. By comparing, we find:

**Table 2:** Test accuracy on UCI datasets for linear classifiers

Datasets	SVM			LSSVM			SRSVM			MPCSVM			HPCSVM	
	Accuracy	Time (s)	*p* value	Accuracy	Time (s)	*p* value	Accuracy	Time (s)	*p* value	Accuracy	Time (s)	*p* value	Accuracy	Time (s)
Hepatitis (155 × 19)	83.30 ± 6.64	0.06	0.6983	83.99 ± 8.98	0.02	0.8315	84.99 ± 8.25	0.07	0.5145	84.61 ± 7.11	0.09	0.9033	*85.24* ± *6.98*	0.06
Heartstatlog (270 × 13)	80.00 ± 5.16	0.16	0.2433	84.07 ± 6.04	0.03	0.8072	84.44 ± 5.05	0.21	0.8825	*85.19* ± *5.86*	0.23		*85.19* ± *6.42*	0.17
Teaching (151 × 5)	68.85 ± 2.80	0.06	0.2216	72.54 ± 5.36	0.03	0.8757	72.94 ± 5.36	0.07	0.8238	72.85 ± 5.31	0.07	0.8596	*73.63* ± *6.65*	0.05
Haberman (306 × 3)	73.53 ± 0.49	0.25	0.4298	74.50 ± 2.57	0.04	0.7847	*75.50* ± *2.57*	0.28	0.8765	75.47 ± 3.55	0.26	0.9067	75.15 ± 3.86	0.17
Breast (683 × 9)	96.04 ± 3.68	1.87	0.5185	96.05 ± 1.95	0.22	0.2999	97.19 ± 1.42	2.18	0.9124	97.21 ± 1.88	2.29	0.9009	*97.36* ± *1.36*	1.59
BUPA (345 × 6)	67.53 ± 3.73	0.30	0.2073	68.70 ± 6.19	0.05	0.5306	69.99 ± 4.55	0.32	0.7536	*71.01* ± *4.20*	0.34	1	*71.01* ± *3.43*	0.24
Diabetes (768 × 8)	71.48 ± 2.65	2.54	0.0435	77.61 ± 4.65	0.34	0.8534	75.61 ± 4.65	2.28	0.5216	78.01 ± 3.87	2.40	0.9365	*78.27* ± *5.02*	1.86
Seeds (210 × 7)	95.24 ± 6.10	0.10	0.0037	97.14 ± 2.78	0.03	0.5796	*98.84* ± *2.78*	0.12	0.8215	98.10 ± 1.78	0.14	1	98.10 ± 1.78	0.10
Sonar (208 × 60)	72.16 ± 9.52	0.10	0.3209	77.34 ± 8.47	0.02	0.8122	78.34 ± 7.73	0.08	0.9354	*79.74* ± *7.56*	0.16	0.8664	78.78 ± 8.12	0.10
Parkinsons (195 × 22)	84.09 ± 1.92	0.08	0.0191	88.23 ± 1.03	0.02	0.5307	88.53 ± 1.03	0.14	0.6346	88.27 ± 3.69	0.13	0.6874	*89.25* ± *2.96*	0.09
Spect (267 × 44)	79.78 ± 0.70	0.25	0.0687	79.40 ± 0.19	0.04	0.0438	82.40 ± 0.19	0.28	0.8765	*83.90* ± *3.61*	0.27	0.7601	83.14 ± 3.13	0.18
Ionosphere (351 × 33)	87.75 ± 2.33	0.45	0.4902	86.61 ± 1.16	0.07	0.0959	88.86 ± 1.41	0.50	0.9645	*90.31* ± *3.32*	0.40	0.4913	88.89 ± 2.12	0.28
Heartcancer (303 × 14)	96.33 ± 7.33	0.16	0.3466	92.09 ± 1.89	0.03	0.0000	98.09 ± 1.78	0.25	0.5024	*100* ± *0.00*	0.29	NaN	*100* ± *0.00*	0.21
Heart_diseas e(294 × 13)	72.79 ± 6.84	0.27	0.0326	82.33 ± 4.19	0.04	0.6854	83.00 ± 3.82	0.30	0.8456	83.35 ± 5.04	0.26	0.9258	*83.69* ± *4.95*	0.19
Fertility (100 × 9)	86.29 ± 4.93	0.02	0.3105	88.08 ± 1.94	0.01	0.6001	90.08 ± 1.98	0.04	0.6570	*90.18* ± *4.04*	0.04	0.6982	89.13 ± 3.34	0.03
Ech_diogram (131 × 10)	87.00 ± 7.04	0.05	0.5075	88.39 ± 6.69	0.03	0.7137	89.39 ± 5.96	0.07	0.9035	*89.96* ± *4.83*	0.06	1	*89.96* ± *4.83*	0.05
Balancescale (576 × 4)	95.49 ± 1.77	0.89	0.4746	95.32 ± 2.08	0.17	0.4419	*96.49* ± *1.57*	1.15	0.9654	96.36 ± 0.83	1.20	0.9986	96.36 ± 1.49	0.83
WPBC (198 × 34)	78.27 ± 5.00	0.10	0.2938	80.36 ± 4.09	0.03	0.5125	82.36 ± 4.67	0.11	0.9941	82.37 ± 4.34	0.14	0.9971	*82.38* ± *5.35*	0.10
WDBC (569 × 30)	93.51 ± 5.13	0.87	0.0000	96.13 ± 1.89	0.18	0.0994	*98.13* ± *2.24*	1.13	0.9235	98.07 ± 0.86	1.32	1	98.07 ± 0.86	0.93
Vertebral (310 × 6)	85.48 ± 2.89	0.25	0.4520	85.16 ± 4.26	0.04	0.4731	86.12 ± 4.52	0.30	0.7538	*87.10* ± *3.38*	0.28	1	*87.10* ± *2.89*	0.21
Australian (690 × 14)	85.51 ± 1.62	2.49	0.2854	86.09 ± 1.12	0.20	0.4450	86.87 ± 2.15	2.25	0.8856	86.96 ± 1.87	1.89	0.9218	*87.11* ± *2.28*	1.55
BTSC (748 × 4)	75.55 ± 2.52	2.51	0.0000	77.27 ± 0.68	0.32	0.1793	77.54 ± 1.03	2.28	0.2058	79.28 ± 2.92	2.08	0.8968	*79.41* ± *2.82*	1.67
Tic_tac_toe (958 × 27)	*98.33* ± *0.39*	2.20	1	*98.33* ± *0.39*	0.26	1	*98.33* ± *0.39*	3.25	1	*98.33* ± *0.39*	5.79	1	*98.33* ± *0.39*	3.00
German (1000 × 24)	74.90 ± 2.22	4.27	0.1839	76.80 ± 2.46	0.28	0.6755	77.10 ± 2.62	6.26	0.7640	77.50 ± 1.38	5.21	0.8997	*77.70* ± *2.74*	4.08
CMC (1473 × 9)	77.39 ± 0.13	11.80	0.0936	77.46 ± 0.52	1.64	0.1467	*78.73* ± *0.43*	14.03	0.9023	78.07 ± 0.96	12.36	0.6478	78.41 ± 1.06	9.06

**Table 3:** Test accuracy on UCI datasets for nonlinear classifiers

Datasets	SVM			LSSVM			SRSVM			MPCSVM			HPCSVM	
	Accuracy	Time (s)	*p* value	Accuracy	Time (s)	*p* value	Accuracy	Time (s)	*p* value	Accuracy	Time (s)	*p* value	Accuracy	Time (s)
Hepatitis (155 × 19)	82.01 ± 9.43	0.10	0.7550	83.34 ± 8.36	0.08	0.9095	83.78 ± 8.12	0.12	0.9643	84.01 ± 7.98	0.16	0.9971	*84.03* ± *8.23*	0.14
Heartstatlog (270 × 13)	82.96 ± 2.46	0.27	0.5264	83.70 ± 6.13	0.21	0.7863	84.70 ± 5.24	0.33	0.9654	84.44 ± 5.32	0.41	0.9218	*84.81* ± *5.02*	0.36
Teaching (151 × 5)	82.14 ± 3.19	0.10	0.7812	82.14 ± 3.19	0.07	0.7812	*83.24* ± *3.45*	0.11	0.8574	82.83 ± 3.59	0.13		82.83 ± 3.59	0.12
Haberman (306 × 3)	73.53 ± 0.48	0.35	0.1056	74.51 ± 3.03	0.26	0.5231	75.32 ± 2.65	0.46	0.8032	75.48 ± 2.98	0.50	0.8693	*75.81* ± *2.45*	0.44
Breast (683 × 9)	95.60 ± 4.54	2.20	0.4155	97.22 ± 1.50	1.34	0.6948	97.62 ± 1.24	3.26	0.9544	97.51 ± 1.36	3.51	0.8913	*97.66* ± *1.56*	3.01
BUPA (345 × 6)	71.01 ± 3.30	0.46	0.0602	73.33 ± 2.67	0.42	0.2547	74.43 ± 2.49	0.48	0.5642	75.36 ± 2.75	0.68	0.8832	*75.65* ± *2.66*	0.65
Diabetes (768 × 8)	72.39 ± 1.86	2.57	0.0384	77.48 ± 4.65	1.68	0.7826	76.48 ± 4.26	2.86	0.5145	78.01 ± 5.01	3.89	0.9104	*78.40* ± *4.48*	3.44
Seeds (210 × 7)	95.95 ± 3.10	0.17	0.1614	96.19 ± 2.43	0.13	0.7599	*96.24* ± *2.10*	0.20	0.5462	96.19 ± 1.17	0.25	0.6666	96.01 ± 1.78	0.23
Sonar (208 × 60)	88.47 ± 6.96	0.17	1	88.47 ± 6.96	0.13	1	*89.59* ± *6.56*	0.21	0.7459	88.47 ± 6.96	0.28		88.47 ± 6.96	0.23
Parkinsons (195 × 22)	87.21 ± 2.68	0.14	0.0016	93.87 ± 2.01	0.12	0.2447	94.28 ± 2.26	0.18	0.5568	*95.95* ± *2.54*	0.24	0.9888	95.92 ± 2.58	0.19
Spect (267 × 44)	79.79 ± 4.06	0.27	0.2639	82.40 ± 4.94	0.22	0.7521	83.40 ± 4.72	0.35	0.9325	*83.89* ± *5.36*	0.45	0.9181	83.51 ± 4.67	0.42
Ionosphere (351 × 33)	94.31 ± 2.54	0.50	0.2920	95.72 ± 1.29	0.36	0.7860	95.45 ± 2.08	0.58	0.6734	*96.29* ± *1.72*	0.78	0.8215	96.02 ± 1.65	0.67
Heartcancer (303 × 14)	96.38 ± 2.62	0.35	0.0247	95.05 ± 3.12	0.27	0.0131	98.05 ± 2.96	0.46	0.5672	*100* ± *0.00*	0.54	NaN	*100* ± *0.00*	0.46
Heart_disease (294 × 13)	75.85 ± 2.71	0.31	0.0178	79.31 ± 4.76	0.24	0.7579	82.31 ± 4.26	0.38	0.8973	83.00 ± 3.53	0.46	0.9085	*83.33* ± *4.24*	0.40
Fertility (100 × 9)	87.12 ± 4.45	0.05	0.2239	88.18 ± 4.61	0.03	0.5031	88.08 ± 1.94	0.06	0.4589	*90.08* ± *2.87*	0.07		*90.08* ± *2.87*	0.06
Ech-diogram (131 × 10)	87.68 ± 5.25	0.07	0.6181	86.16 ± 5.95	0.06	0.2633	89.24 ± 5.64	0.09	0.8575	89.96 ± 6.41	0.10	0.9951	*89.99* ± *7.20*	0.09
Balancescale (576 × 4)	97.92 ± 1.61	1.34	0.0709	98.79 ± 0.88	0.98	0.1146	99.19 ± 0.82	1.58	0.3678	99.48 ± 0.69	1.89	0.6840	*99.65* ± *0.42*	1.65
WPBC (198 × 34)	79.85 ± 5.15	0.15	0.4901	80.35 ± 6.11	0.13	0.6146	*83.12* ± *4.22*	0.20	0.7236	82.87 ± 3.52	0.24	0.8650	82.36 ± 4.67	0.20
WDBC (569 × 30)	96.14 ± 1.03	1.27	0.0075	98.41 ± 0.87	0.91	0.6163	98.62 ± 0.95	1.32	0.7148	98.59 ± 0.89	2.04	0.8044	*98.77* ± *1.05*	1.71
Vertebral (310 × 6)	85.48 ± 2.28	0.36	0.4774	85.81 ± 3.44	0.27	0.6224	86.41 ± 2.54	0.42	0.8012	86.77 ± 2.58	0.53	0.8894	*87.10* ± *3.68*	0.51
Australian (690 × 14)	85.51 ± 1.62	2.05	0.4526	86.23 ± 2.00	1.37	0.6902	86.78 ± 1.69	2.39	0.8536	86.96 ± 1.28	3.11	0.9451	*87.11* ± *1.82*	2.73
BTSC (748 × 4)	77.01 ± 2.86	2.41	0.2378	78.88 ± 1.91	1.56	0.6675	79.46 ± 2.51	2.76	0.8245	*79.82* ± *2.76*	3.71	0.9504	79.68 ± 3.07	3.33
Tic_tac_toe (958 × 27)	*98.33* ± *0.39*	3.99	1	*98.33* ± *0.39*	2.69	1	*98.33* ± *0.39*	4.73	1	*98.33* ± *0.39*	9.37	1	*98.33* ± *0.39*	5.45
German (1000 × 24)	75.40 ± 2.85	4.56	0.4943	74.40 ± 1.66	2.93	0.2232	76.32 ± 1.87	5.21	0.7435	77.00 ± 1.03	9.35	0.9288	*77.20* ± *2.74*	8.27
CMC (1473 × 9)	77.39 ± 0.13	9.99	0.1728	77.66 ± 0.55	6.17	0.3959	78.46 ± 0.55	2.30	0.9765	78.28 ± 0.88	16.78	0.9246	*78.59* ± *1.08*	15.31

1. *SRSVM versus SVM*: In SRSVM, we adopt the Ward’s linkage clustering to capture the underlying data distribution within classes, and then the structural information is directly embedded into the objective function by the minimization of the compactness between the estimated clusters. For both linear and nonlinear classifiers, we can find that, except for Tic-tac-toe, all the remaining experimental results of SRSVM are much better than that of SVM. In a word, the comparison between these two methods illustrates the prior structural information within classes in the data is effective for classification.
2. *MPCSVM versus SVM*: In MPCSVM, we adopt the MPC trick to extract the discriminative information, then introduce the coresponding regularization term into SVM. For linear classifiers, we can find that, except for Tic-tac-toe, all the remaining experimental results of MPCSVM are better than that of SVM. For nonlinear classifiers, MPCSVM and SVM get the same experimental results on Sonar and Tic-tac-toe, while for other twenty-three datasets, all the experimental results of MPCSVM are superior to that of SVM. Thus, we may safely draw the conclusion, the discriminative information about each constrained pair in data is effective for classification.
3. *MPCSVM versus SRSVM*: For both linear and nonlinear classifiers, there exist sixteen datasets whose experimental results of MPCSVM are better than that of SRSVM, respectively, even if the datasets are not exactly the same in the two cases. Thus, we may safely draw the conclusion, the discriminative information about each constrained pair is more effective than the structures in the data within classes for classification.
4. *HPCSVM versus MPCSVM*: In our HPCSVM, we adopt the HPC trick to extract the discriminative information in data, then introduce the coresponding regularization term into SVM. For linear classifiers, we can find that, HPCSVM and MPCSVM get the same experimental results on nine datasets, while for other sixteen datasets, there are twelve datasets whose experimental results of HPCSVM are better than that of MPCSVM. For nonlinear classifiers, there exist only six datasets whose experimental results of HPCSVM are poorer than that of MPCSVM. So, the comparison of these two methods demenstrates that the discriminative information with high-order relationship between different samples is more reasonable.
5. *HPCSVM versus LSSVM*: For linear classifiers, except for Tic-tac-toe, all the remaining experimental results of HPCSVM are better than that of LSSVM. For nonlinear classifiers, except for Seeds, Sonar and Tic-tac-toe, the remaining twenty-two experimental results of HPCSVM are better than that of LSSVM. Hence, the comparison of these two methods illustrates our proposed HPCSVM is not only better than SVM, but also better than its variants.

From Tables 2, 3, we may safely draw the conclusion, as long as the discriminative information regularization term is adopted, the best results always appear in the last two columns, i.e., experimental results on the twenty-five benchmark datasets demonstrate the discriminative information about each constrained pair in data is more effective than the structural information within classes for classification. Furthermore, we remodify the recently presented MPC regularization term with HPC method, which is expected to acquire the high-order relationship between different samples. And the comparison of the last two columns demonstrates the discriminative information with high-order relationship between several samples is more reasonable than with the pairwise relationship between two samples. In the following paper, we further analyze the statistically significant difference between results and the influence of parameter $k$ in our HPCSVM.

#### Statistical test

In statistics, the Holm–Bonferroni test is a simple method for multiple Student’s *t* test. For this, first, we order the *p* value of each dataset in ascending order as $p_{(1)} ,p_{(2)} ,p_{(3)} ,p_{(4)}$, and denote the associated hypotheses as $H_{(1)} ,H_{(2)} ,H_{(3)} ,H_{(4)}$. For the given significance level $\alpha = 0.05$, let $m$ be the minimal index such that

44 $$p_{(m)} > \frac{\alpha }{4 + 1 - m}$$

Then, the null hypotheses $H_{(1)} , \ldots ,H_{(m - 1)}$ are rejected and $H_{(m)} , \ldots ,H_{(4)}$ are not rejected. In this way, we can find that, for the linear case, among the 100 null hypotheses, there exist 4 hypotheses which are judged that our method has significant advantage over others, while for the nonlinear case, there only exist 2. These test results illustrate that our method is not obviously better than others. However, it is worth mentioning that HPCSVM obtains the better accuracies than the other algorithms on most datasets. This indicates that HPCSVM does not reduce any generalization performance compared with others.

#### Influence of cluster parameter

**Fig. 2:**
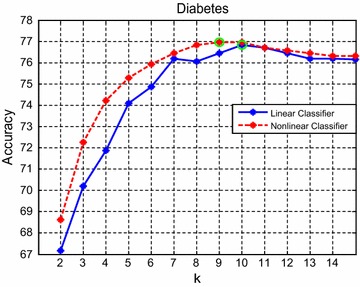
Changes of accuracy as the growth of $k$

Below, in order to investigate the influence of cluster parameter $k$ in our HPCSVM, we perform an experiment on a relatively large dataset, i.e., Diabetes. And the value of $k$ ranges from 2 to 15. The comprehensive experimental results are shown in Fig. 2, which shows the tendency of testing precision as the growth of $k$. Note that, for both linear and nonlinear cases, the testing precision first increases and then decreases as the growth of $k$. The main reason may be, a too small $k$ could easily remove some useful information, whereas, a too large $k$ could introduce some noise points, and both of the two cases could reduce the prediction accuracy. Thus, an appropriate parameter $k$ is very important.

## Conclusion

As we know, the potential structural information of the training data is ignored by SVM. In order to mitigate this shortcoming, in this paper, we present a novel algorithm termed as HPCSVM to improve the generalization performance of SVM. More specifically, on the one hand, we could acquire the high-order relationships between different samples by hypergraph learning, on the other hand, we present a more reasonable discriminative regularization term by combining the discrimination metric and hypergraph learning together, at last, we introduce this novel regularization term into SVM to adjust the optimal separating hyperplane which is obtained by SVM. As expected, the novel model yields better generalization performance than SVM and its variants. However, it has more tuning parameters, so, effective model selection for the new method is an open research area.
